# Species composition and seasonal dynamics of aphid parasitoids and hyperparasitoids in wheat fields in northern China

**DOI:** 10.1038/s41598-017-14441-6

**Published:** 2017-10-25

**Authors:** Fan Yang, Lei Xu, Yue-Kun Wu, Qian Wang, Zhi-Wen Yao, Vladimir Žikić, Željko Tomanović, Mar Ferrer-Suay, Jesús Selfa, Juli Pujade-Villar, Michael Traugott, Nicolas Desneux, Yan-Hui Lu, Yu-Yuan Guo

**Affiliations:** 1 State Key Laboratory for Biology of Plant Diseases and Insect Pests, Institute of Plant Protection, Chinese Academy of Agricultural Sciences, Beijing, 100193 China; 2 Faculty of Sciences and Mathematics, Department of Biology and Ecology, University of Niš, Višegradska 33, 18000 Niš, Serbia; 3 University of Belgrade, Faculty of Biology, Institute of Zoology, Department of Invertebrate Zoology and Entomology, Belgrade, 11000 Serbia; 4 Universitat de València, Facultat de Ciències Biològiques, Departament de Zoologia, València, 46100 Spain; 5 Universitat de Barcelona, Facultat de Biologia, Departament de Biologia Animal. Avda. Diagonal 645, 08028 Barcelona, Spain; 6 Mountain Agriculture Research Unit, Institute of Ecology, University of Innsbruck, 6020 Innsbruck, Austria; 7 INRA (French National Institute for Agricultural Research), Université Côte D’Azur, CNRS, UMR 1355-7254, Institut Sophia Agrobiotech, 400 Route des Chappes, 06903 Sophia Antipolis, France

## Abstract

Parasitoids are important natural enemies of aphids in wheat fields of northern China, and interest in them has increased in recent years. However, little is known regarding parasitoids of wheat aphids, which has hindered the study and understanding of aphid-parasitoid interactions. In the present study, three primary parasitoids and 15 hyperparasitoids were collected in wheat fields during a 2-year survey in northern China (2014, 2015) and a 2-year investigation at Langfang, Hebei Province (2015, 2016). Among them, *Aphidius uzbekistanicus* Luzhetski was found most frequently among the primary parasitoids, while *Pachyneuron aphidis* (Bouché) dominated the hyperparasitoid community. Investigation of the dynamics of wheat aphids and parasitoids revealed that the primary parasitoids appeared early in the growing period and that the hyperparasitoids appeared later. Analysis of the seasonal dynamics revealed that growth of the parasitoid population followed that of the aphid population and that the parasitism rates were highest in the late growing period.

## Introduction

In northern China, four common aphid species, *Sitobion avenae* (Fabricius), *Schizaphis graminum* (Rondani), *Metopolophium dirhodum* (Walker), and *Rhopalosiphum padi* (L.), dominate the herbivore community in fields of winter wheat, *Triticum aestivum* L.^1,2^. Their large numbers and the serious damage they cause leads to great losses in wheat yield each year. For example, at more than 2,000 wheat aphids per 100 plants, kernel weight (for 1000 kernels) can be reduced by up to 45%^3^. Fortunately, these aphids are attacked by a suite of natural enemies, including parasitoid wasps, which are an important component of the wheat field ecosystem. The parasitism rates of wheat aphids can reach 70–80%^4^ or even 90% in some fields^1^. However, we still lack a comprehensive understanding of the parasitoid community in wheat fields and of the efficiency of these parasitoids in controlling these pests.

Aphid-parasitoid relationships have been extensively documented, with topics including host specificity^5,6^, trophic relationships^7^, community structure^8^, foodwebs^9,10^, and the influences of factors such as landscape complexity^9,11,12^, agricultural intensification^10^ and farming practices^13^. Traditional study methods, such as rearing and dissection, are time- and labor-intensive. Furthermore, specialists in morphological identification are required, and some taxa are difficult to distinguish based on the very restricted number of reliable morphological characters in parasitoids^14–17^, in case of cryptic species^18^. An alternative to that are molecular approaches to analyze parasitoid DNA within their hosts which can avoid shortcomings of traditional approaches^17–22^; this approach can also reveal cryptic relationships in some cases^23–25^.

To develop such a molecular detection technique for Chinese cereal aphid parasitoids, we first compiled information on wheat aphid parasitoids to familiarize ourselves with these species. Most of the literature on wheat aphid parasitoids in China is focused on primary parasitoids, particularly species of Aphidiinae^1,2,26–32^. However, aphid^33^ and primary parasitoid^33,34^ populations can be influenced by hyperparasitoids, of which reports are mostly lacking. Most records are from the 1990s, with five hyperparasitoid species in this system being recorded in northern China and four in central China. These include *Aphidencyrtus* (*Syrphophagus*) *aphidivorus* (Mayr), *Dendrocerus carpenteri* (Curtis), *Pachyneuron aphidis* (Bouché), and other species that could only be classified to genus^1,35,36^. Recently, Zhao *et al*. (2011) and Hu *et al*. (2016) recorded nine hyperparasitoid species in northwestern China and provided detailed data on the abundance for each species; however, some also could not be identified to the species level^37,38^.

To understand the biological control effect these aphid parasitoids have, it is important to understand their relative efficiency, which would allow conservation efforts to target the most important species. How species from different trophic levels interact with each other and how they affect the food web as a whole, such as the effect of hyperparasitoids on the biological control efficiency, are still unclear. Moreover, comprehensive information about the aphid-parasitoid species is missing and the dominant species of aphid hosts, primary parasitoids, and hyperparasitoids at different growing periods is still unknown.

**Figure 1:**
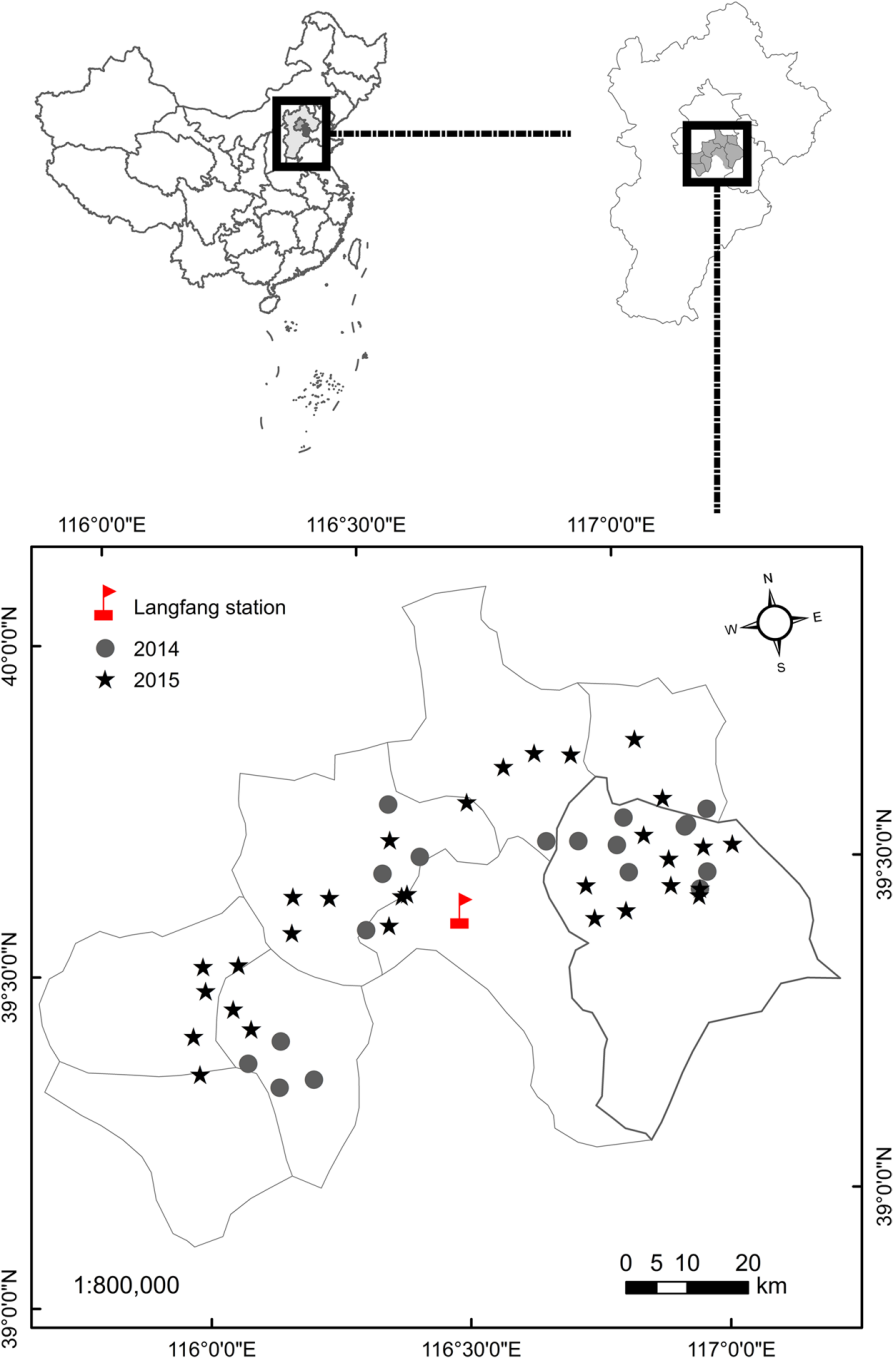
The distribution of the wheat fields used for sampling of aphid and parasitoids in northern China in 2014 (circles) and 2015 (pentagrams). The site maps were created by using Geostatistical Analyst Extension of ArcGIS 10.2 (ESRI, 2014). The flag represents the Langfang Experimental Station, IPP, CAAS.

**Figure 2:**
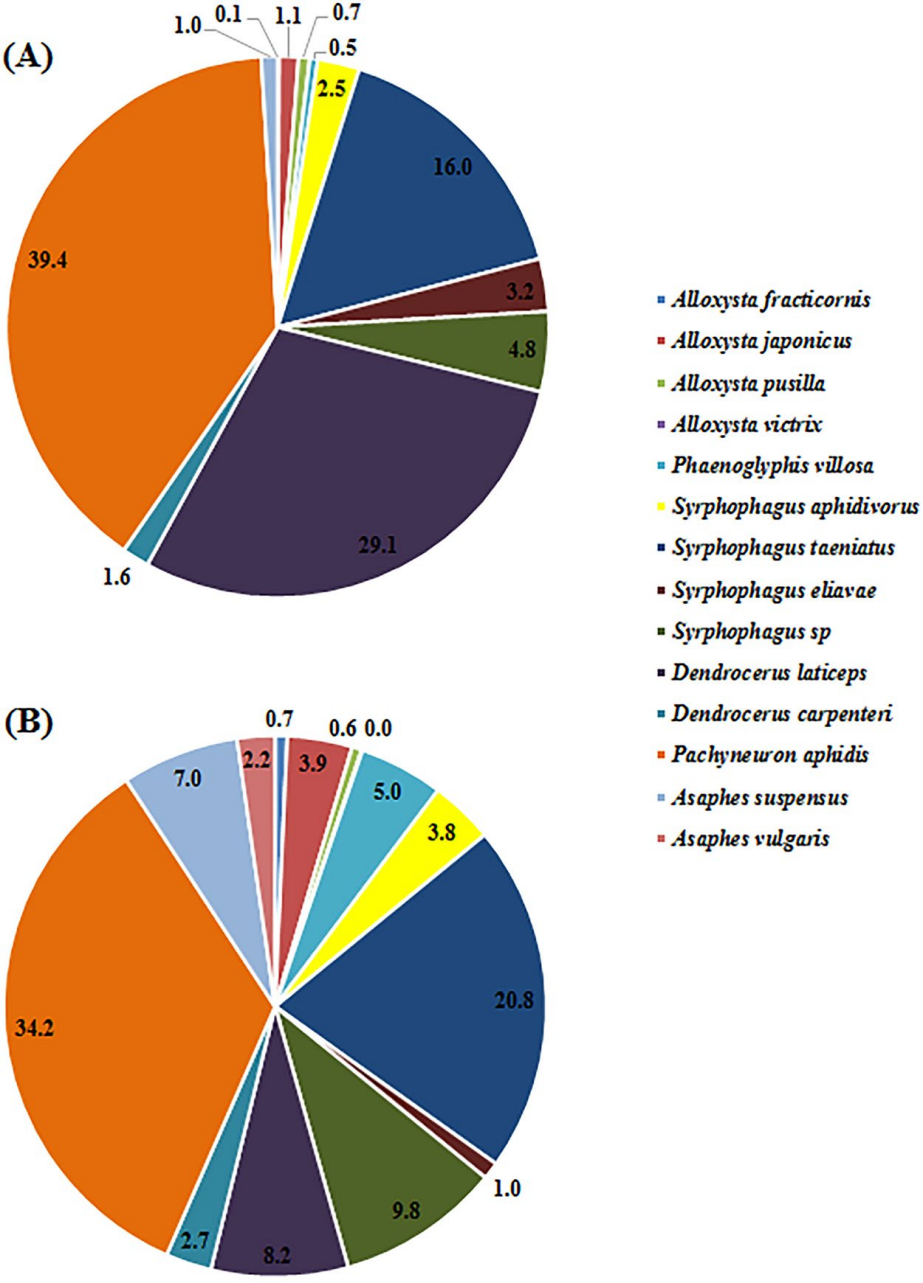
Species composition and relative percentages of hyperparasitoids in wheat fields in northern China, 2014/2015. (**A**) The relative species composition of hyperparasitoid of individuals collected from 18 wheat fields in 2014, and (**B**) the composition of individuals collected from 30 wheat fields in 2015.

Here, we investigated (1) the parasitoid species composition in 18 wheat fields in 2014 and 30 fields in 2015 in northern China (Figs 1 and 2) the population dynamics of wheat aphids and their parasitoids in Langfang, Hebei Province from 2015–2016. This work will support future research on the species characterization and community composition of wheat aphid parasitoids in China.

## Results

### Parasitoid species composition in commercial wheat fields in northern China

In 2014, 460 primary parasitoid individuals from two species and 1,047 hyperparasitoid individuals belonging to 12 species emerged from the mummies collected from 18 wheat fields. In 2015, 1,276 primary parasitoid individuals belonging to three species and 2,048 hyperparasitoid individuals belonging to 14 species emerged from the mummies collected within 30 wheat fields.

Two primary parasitoid species, *Aphidius uzbekistanicus* Luzhetski and *Aphidius gifuensis* (Ashmead), were found in 2014, and a third primary parasitoid species, *Aphidius ervi* Haliday, was found in 2015. *Aphidius uzbekistanicus*, the most abundant primary parasitoid, represented 72.17% and 86.21% of the primary parasitoid individuals in 2014 and 2015, respectively. The percentage of the second-most abundant species in the overall catches, *A*. *gifuensis*, decreased from 27.83% in 2014 to 12.2% in 2015. The 21 *A*. *ervi* individuals collected in 2015 represented only 1.65% of that year’s primary parasitoids that were collected.

The hyperparasitoid guild was more diverse, consisting of 14 species: *Alloxysta fracticornis* (Thomson), *A*. *japonicus* (Ashmead), *A*. *pusilla* (Kieffer), *A*. *victrix* (Westwood), *Asaphes suspensus* (Nees), *A*. *vulgaris* Walker, *Dendrocerus carpenteri*, *D*. *laticeps* (Hedicke), *Pachyneuron aphidis*, *Phaenoglyphis villosa* (Hartig), *Syrphophagus aphidivorus*, *S*. *eliavae* Japoshvili, *S*. *taeniatus* (Förster), and *Syrphophagus* sp. In this guild, *P*. *aphidis* was by far the most abundant species, representing 39.45% and 34.23% of the hyperparasitoids collected in 2014 and 2015, respectively. Two species, *A*. *victrix* and *A*. *vulgaris*, were only observed in 2015. The second-most abundant species changed from *D*. *laticeps* (29.13%) in 2014 to *S*. *taeniatus* (20.80%) in 2015. From 2014 to 2015, the relative catch of *A*. *pusilla* decreased slightly from 0.67% to 0.59%, that of *S*. *eliavae* decreased from 3.15% to 1.03% and that of *D*. *laticeps* decreased from 29.13% to 6.98%. In contrast, the relative catches of the remaining hyperparasitoid species increased from 2014 to 2015. All had relative catches under 10% except for *P*. *aphidis*, *D*. *laticeps* and *S*. *taeniatus* (Fig. 2).

### Seasonal dynamics of wheat aphids and parasitoids in wheat fields at the Langfang site

Among four wheat aphid species, *S*. *avenae* was dominant and accounted of 89.80% of all aphids collected in 2015 and 72.99% in 2016. The least abundant aphid species in 2015 was *M*. *dirhodum* (0.01%), but its proportion increased to 11.81% in 2016. There was also an increase of the relative *R*. *padi* population, from 9.26% (2015) to 14.30% (2016). *S*. *graminum* had similar proportions in both years, 0.93% in 2015 and 0.91% in 2016.

A total of 579 parasitoid individuals belonging to 16 species were collected from the three wheat field plots at Langfang in 2015, and 981 parasitoids belonging to 14 species were collected in 2016. The species compositions were similar to those in northern China, although one new species, *Alloxysta consobrina* (Zetterstedt), was found in 2015.

**Figure 3:**
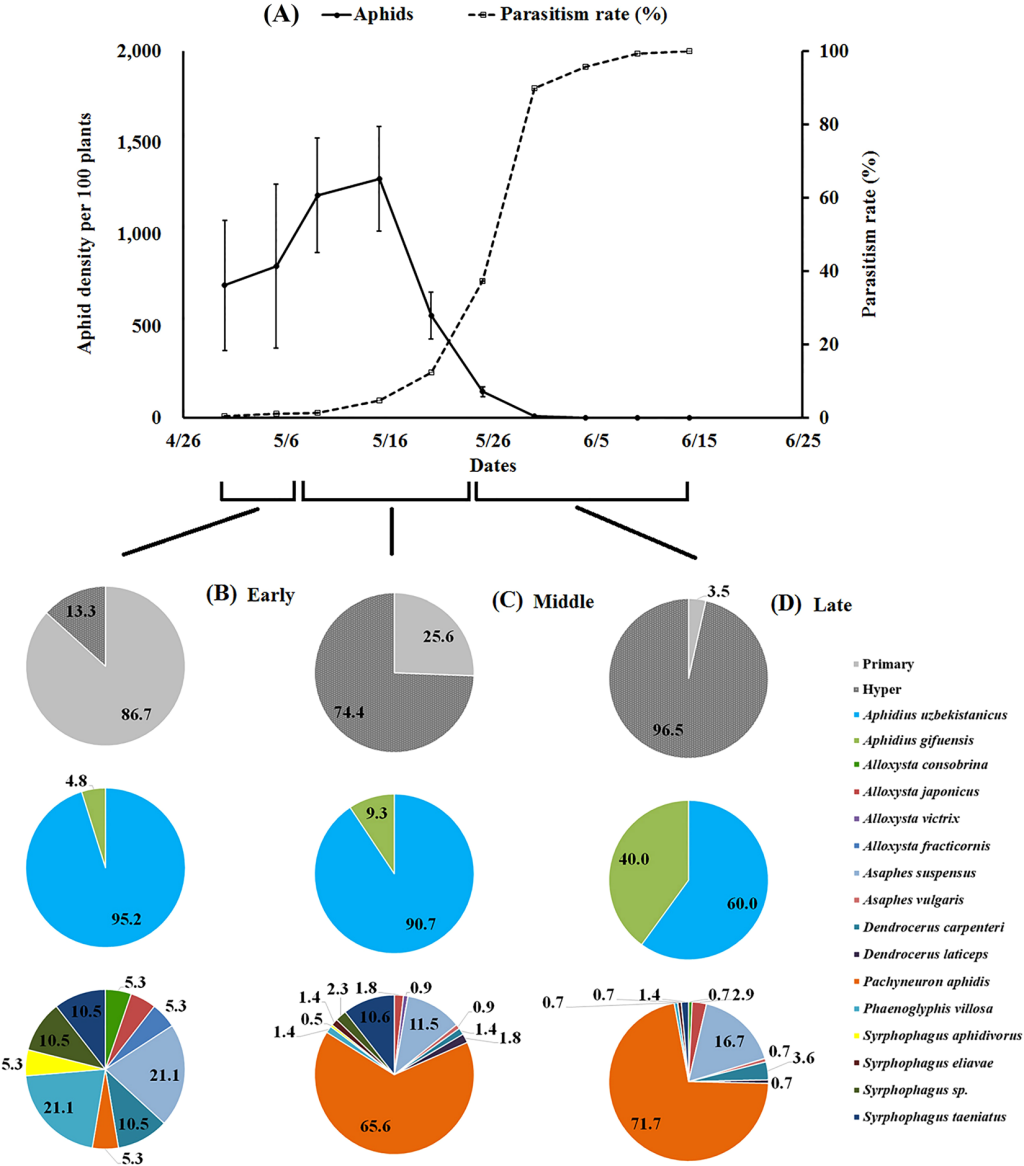
Wheat aphid population dynamics, parasitism rates, and wheat aphid parasitoid diversity and composition in Langfang, Hebei (2015). (**A**) Wheat aphid population dynamics and parasitism rates in 2015, (**B**) the proportion of wheat aphid primary parasitoid and hyperparasitoid and their diversity and composition at early growing period (4/30-5/9), (**C**) the proportion of wheat aphid primary parasitoid and hyperparasitoid and their diversity and composition at middle growing period (5/10-5/25), (**D**) the proportion of wheat aphid primary parasitoid and hyperparasitoid and their diversity and composition at late growing period (5/26-6/14).

**Figure 4:**
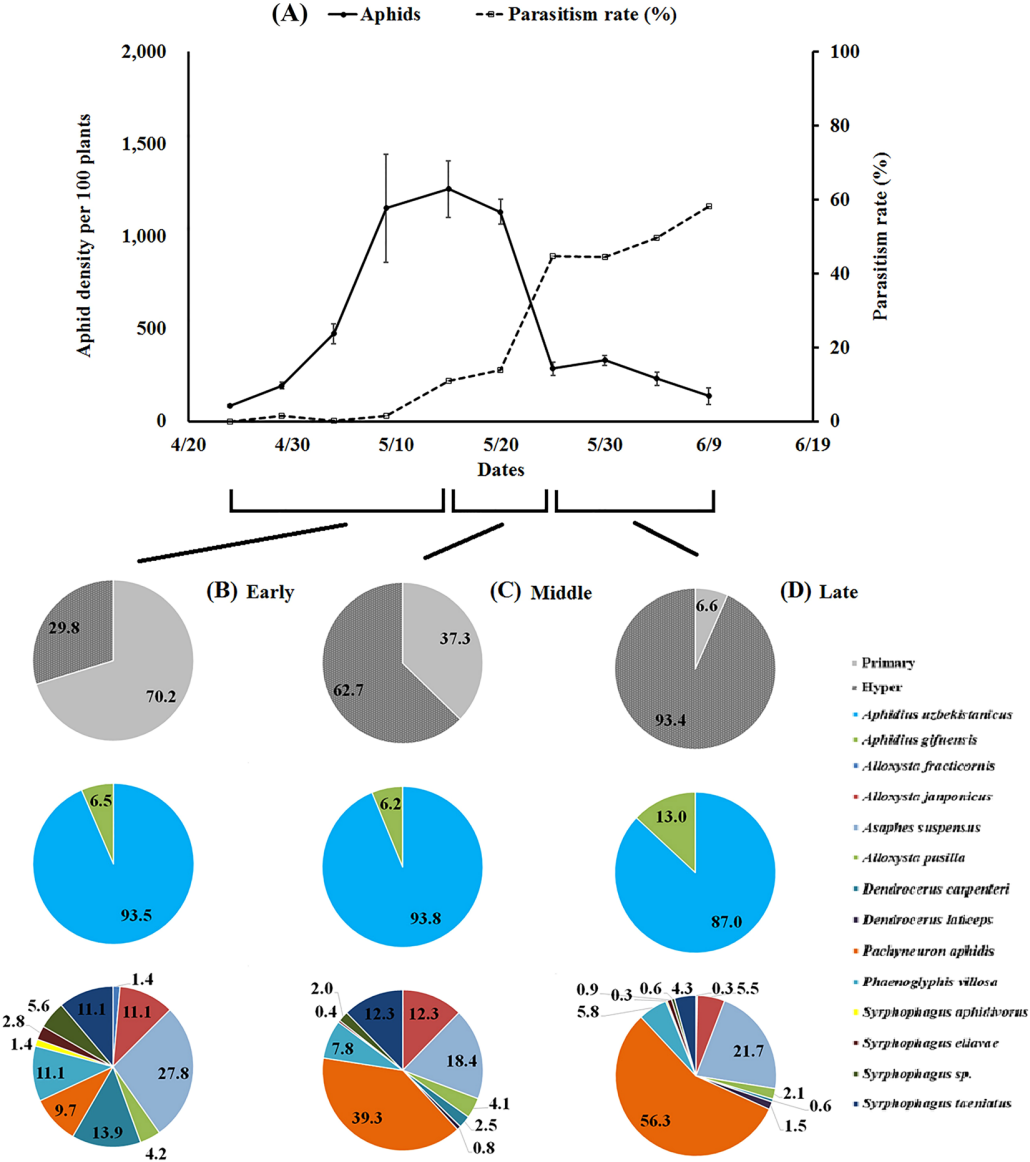
Wheat aphid population dynamics, parasitism rates, and wheat aphid parasitoid diversity and composition in Langfang, Hebei (2016). (**A**) Wheat aphid population dynamics and parasitism rates in 2016, (**B**) the proportion of wheat aphid primary parasitoid and hyperparasitoid and their diversity and composition at early growing period (4/24-5/15), (**C**) the proportion of wheat aphid primary parasitoid and hyperparasitoid and their diversity and composition at middle growing period (5/16-5/25), (**D**) the proportion of wheat aphid primary parasitoid and hyperparasitoid and their diversity and composition at late growing period (5/26-6/9).

The aphid population increased from the start of investigation and peaked on May 15 in both 2015 and 2016. In 2015, the aphid density decreased thereafter from 1,304 to 558 per 100 plants. In 2016, a pronounced decrease occurred after the May 25 sampling, from 1,134 to 285 per 100 plants. The parasitism rate increased throughout the growing season in both years, and it reached 100% on June 14 in 2015 when aphid densities were very low. The highest parasitism observed during 2016 was 58.12% on June 9, and there was a slight decrease observed from May 30 from 44.72% to 44.37% (Figs 3 and 4).

The ratio of primary/hyper parasitoids varied significantly among different growing periods in both years (2015: χ^2^ = 244.15, df = 2, *P* < 0.0001; 2016: χ^2^ = 259.15, df = 2, *P* < 0.0001). The primary parasitoid proportion of the total parasitoids decreased from 86.71% (early period) to 3.50% (late period) in 2015 and from 70.25% (early period) to 6.57% (late period) in 2016. For the ratio of different primary species, there were no significance changes during the whole growing period in 2016, which was contrary to 2015 (2015: χ^2^ = 9.41, df = 2, *P* = 0.0091; 2016: χ^2^ = 1.52, df = 2, *P* = 0.4669). *Aphidius uzbekistanicus* dominated the primary parasitoids, being at least 87.0% of total in 2015 and 60.0% in 2016. The proportion of various hyperparasitoids during different growing periods showed significant differences in both years (2015: χ^2^ = 119.72, df = 26, *P* < 0.0001; 2016: χ^2^ = 115.78, df = 22, *P* < 0.0001). In the early period, *A*. *suspensus* (21.05%) was the most abundant hyperparasitoid in both years, but *P*. *villosa* had the same percentage in 2015. For the remainder of the growing period, *P*. *aphidis* was the most abundant hyperparasitoid, accounting for 71.74% of the hyperparasitoid group in the late growing period in 2015. In addition, two hyperparasitoid species, *A*. *consobrina* and *A*. *vulgaris*, were only observed in 2015.

## Discussion

Our study, spanning a 2-year period, represents the full investigation of the diversity and community composition of wheat aphid parasitoids in northern China. Among the 13 published studies that record wheat aphid parasitoids in detail, only three were conducted in northern China^1,2,27^. The primary parasitoids recorded by these studies consisted of six species: *Aphidius avenae* Haliday, *A*. *gifuensis*, *A*. *ervi*, *Ephedrus persicae* Froggatt, *E*. *plagiator* (Nees), and *Praon* sp.^25^; one additional species, *Lipolexis gracilis* (Förster), was present the following next year by the same authors as before^1^. A study conducted in 2007 found primary parasitoid species of the genera *Aphidius*, *Diaeretiella*, *Ephedrus*, and *Lysiphlebus* ^2^. Compared with these previous works, the present study found fewer primary parasitoid species, all in the genus Aphidius: *Aphidius uzbekistanicus*, *A*. *gifuensis* and *A*. *ervi*. Only one previous work mentioned *A*. *uzbekistanicus* ^29^; and it appears not to an uncommon primary aphid parasitoid in wheat fields in China. In contrast, it is a consistent and important member of cereal aphid parasitoid communities in the Palaearctic^39,40^. However, in the present study, *A*. *uzbekistanicus* replaced *A*. *avenae* as the most abundant of primary parasitoid species in China^37,38^. Interestingly, no *A*. *avenae* were found in this study, a finding that brings into question the results of previous studies. Either *A*. *avenae* was misidentified in previous work or *A*. *uzbekistanicus* has replaced *A*. *avenae* in northern Chinese cereal crops for competitive reasons. One new species, *A*. *ervi* was found in 2015. The primary parasitoid collection in 2015 consisted of more than twice as many samples that in 2014. This expanded collection has likely increased the odds of discovering new species.

Hyperparasitoids have received less attention than primary parasitoids in past studies. Only five species have been reported for northern China, belonging to four families: Figitidae, Encyrtidae, Megaspilidae, and Pteromalidae. Of these, *P*. *aphidis* was the only species identified to species level; the remaining taxa were identified only to the genus level^1^. The relatively comprehensive species descriptions were generated based on collections in northwestern China which was different from our background^37,38,41^. We recollected most of these hyperparasitoid species in our study and identified nine species, belonging to four families: one species each from Encyrtidae and Megaspilidae, three species from Figitidae, and four species from Pteromalidae. *Aphidencyrtus* (*Syrphophagus*) *aphidivorus*. Among them, *D*. *carpenteri* (Megaspilidae), *A*. *suspensus*, *A*. *vulgaris* and *P*. *aphidis* (Pteromalidae) also appeared in our survey. In previous studies, *Alloxysta* spp. could not be classified to species^37,38^. In contrast, we identified five *Alloxysta* species: *A*. *consobrina*, *A*. *fracticornis*, *A*. *japonicus*, *A*. *pusilla* and *A*. *victrix*. In addition, three *Syrphophagus* species, i.e., *S*. *eliavae*, *S*. *taeniatus*, and *Syrphophagus* sp., along with *D*. *laticeps* and *P*. *villosa* were identified in the study, for a total of 15 hyperparasitoid species.

Wheat aphid population densities were low during this study early on and peaked midway through the growing period. Parasitism rates increased throughout almost the entire growing season and followed the trends in growth and decline of the aphid populations, a typical seasonal dynamic of aphid-parasitoids^10^. In general, parasitoids seemed to have suppressed aphid populations during the late growing period. The primary parasitoids appeared during the early growing season, and hyperparasitoids appeared in the late growing season (Figs 3 and 4). In the early growing period, the parasitism rates were quite low and grew slowly such that the primary parasitoids appeared unable to prevent the increase in aphid numbers until late May, when the hyperparasitoids emerged^13^. This pattern was also apparent from the aphids’ and parasites’ seasonal dynamics during the middle growing period, when the parasitism rates were too low to stop the aphid population’s growth. Hyperparasitoids may disrupt the biological control mediated by primary parasitoids and influence the latter’s aphid control efficiency^33,42,43^. In some cases, hyperparasitoids can promote aphid suppression by stabilizing insect-parasitoid dynamics^42,44^, an interesting relationship which needs further study. Considering the delay in the control effect of parasitoids, efforts to protect parasitoids should be made in the early spring (using habitat strips and non-crop planting patterns) to ensure successful colonization. The use of pesticides should be reduced or avoided during the early growing season to protect parasitoid populations.

The aphid-parasitoid system model is a simple, common pest-natural enemy system model^8,10,11,45,46^. However, in our study system, the relationships between specific species of aphids and parasitoids remain unclear. In some studies, parasitoids have been reared with prior knowledge of their target aphid species^9,47^, but in many cases this species-specific relationship is unknown. To this end, barcoding techniques or PCR detection can provide more robust species identification, including the identification of cryptic species^47–50^. However, regardless of the methodological approach, a comprehensive understanding of the parasitoids species and composition will facilitate research on aphid-parasitoid relationships^23^.

## Materials and Methods

### Wheat aphid parasitoid species composition

#### Study area

The study was carried out in Beijing City and Tianjin City, Hebei Province, northern China. This agricultural landscape consists of various habitats, including crops, fallow land, grassland and forest. Wheat is the dominant crop during winter and spring. A total of 18 fields and 30 fields were investigated in 2014 and 2015, respectively (Fig. 1). All fields were randomly selected, and each field was > 1 ha. The distance between fields ranged between 1.5 km and 95.7 km. Sampling was conducted between May 18 and June 5 in 2014, and from May 16 to May 31 in 2015. There were three sampling dates per field each year, with a 7–10-day interval between sampling dates.

#### Parasitoid sampling method

A minimum of 150 aphid mummies were collected at five randomly selected sampling points per wheat field at each sampling date. Sampling sites were separated by at least 5 m, and were at least 10 m away from the field edge. All collected mummies were stored individually in 1.5 mL reaction tubes and brought to the laboratory for parasitoid rearing at 25 ± 1 °C, a photoperiod of 16:8 h (L:D), and 65–75% relative humidity. The lids of the reaction tubes were opened and the opening plugged with absorbent cotton to allow ventilation and prevent the escape of emerged parasitoids. Samples were inspected daily for parasitoids emergence, and emerged parasitoids were stored at 4 °C in 1.5-mL centrifuge tubes filled with 75% ethanol for identification. The number of emerged parasitoids from each sampling field was recorded (Table S1).

#### Parasitoid identification

Most of the reared aphid parasitoids were identified based on morphological characteristics. In addition, part of the mitochondrial cytochrome *c* oxidase subunit one (COI) and the 16 S gene DNA were sequenced from a subsample of the emerged parasitoid specimens to confirm the species identification. DNA was extracted from the parasitoids using a non-destructive DNA extraction (see below). A detailed description of the identification methods and the number of sequences per parasitoid species is provided in Tables S2–3. Emerged parasitoids were morphologically identified under a fluorescent stereomicroscope (SZX16, Olympus, Japan) using specific identifying characteristics. Photographs of the key identification characters were taken using a polarizing microscope (DM2500, Leica, Germany) and a digital camera (EOS 505D, Canon, Japan). Examples of such photographs are provided for different parasitoid species in the supplementary material (Figure S1).

The DNA of three specimens of each parasitoid species was extracted using a non-destructive extraction method. Air-dried parasitoids were submerged in 180 μL Buffer ATL and 20 μL Proteinase K (QIAGEN DNeasy kit, QIAGEN, Germany) at 56 °C for 2 hours and then removed before performing the subsequent steps. The primers LepF1 (5′-3′ ATT CAA CCA ATC ATA AAG ATA TTG G) and LepR1 (5′-3′ TAA ACT TCT GGA TGT CCA AAA AAT CA)^51^ were used to amplify an approximately 700-bp region of the COI gene for each parasitoid. The primers LR-N13398 (bee version) (5′-3′ CAC CTG TTT ATC AAA AAC AT)^52^ and LR-J12888 (supplementary honeybee) (5′-3′ TCG ATT TGA ACT CAR ATC ATG TA)^53^ were used to amplify an approximately 450-bp region of the 16S gene. Amplification of an approximately 1000-bp sequence of the 18S gene was performed using the universal primers 18SL0001 (5′-3′ TAC CTG GTT GAT CCT GCC AGT) and 18SR1100 (5′-3′CGA CGA TCC AAG AAT TTC AC)^54^. PCR followed the universal primer amplification protocols described in Yang *et al*.^55^. PCR products were visualized using a UV transilluminator (Universal Hoodα, Bio-Rad, USA) after electrophoresis at 180 V in a 1% agarose gel for approximately 12 min. Those PCR products which showed a single band of the expected fragment size were sent for sequencing in both forward and reverse directions (Sangon Biotech, Beijing, China). The sequences were then edited and checked using BLAST (Basic Local Alignment Search Tool, NCBI National Center for Biotechnology Information, https://blast.ncbi.nlm.nih.gov/Blast.cgi).

### Seasonal dynamics of wheat aphids and parasitoids

#### Study area

In 2015 and 2016, the seasonal dynamics of aphids and parasitoids was recorded in three winter wheat (var. Zhongmai 175, Institute of Crop Sciences, CAAS, Beijing, China) plots located at the Langfang Experimental Station (GPS coordinates 116.6°E, 39.5°N), Institute of Plant Protection (IPP) of the Chinese Academy of Agricultural Sciences (CAAS) at Langfang. The three plots were 27 × 27 m and separated by more than 5 m from each other. No insecticides or herbicides were applied, and standard agronomic practices for northern China were used during the trial, including fertilization (375 kg/ha urea, 225 kg/ha phosphorus diamine, 150 kg/ha potassium sulfate), regular tillage, and weeding.

#### Population sampling

Mummy parasitoid samples were collected from the three plots, including five 1 × 1 m^2^ sampling points per plot. The densities of aphids and mummies were recorded every five days between April 30 and June 15 in 2015 and between April 24 and June 9 in 2016. On each sampling date, the numbers of aphids and aphid mummies were counted on 20 randomly selected wheat plants during the collection of mummy samples to calculate aphid and mummy densities^8,9^. The storage of the mummy samples, the rearing of parasitoids and their identification were as described above.

### Data analysis

Parasitism rates were calculated by dividing the number of aphid mummies by the sum of mummies and living aphids combined. A Chi-squared test (proc freq) was used to assess the differences among parasitoid taxa, including the two primary parasitoids and various hyperparasitoids on wheat collected from different growing periods in 2015 and 2016 (Table S4). Analyses were performed by using SAS 9.3 software (SAS Institute Inc., Cary, USA).

## Electronic supplementary material

Supplementary information
