# Supplementary material for: Species composition and seasonal dynamics of aphid parasitoids and hyperparasitoids in wheat fields in northern China
Source: Sci Rep. 2017 Oct 25;7:13989. doi: 10.1038/s41598-017-14441-6 (PMC5656665; doi:10.1038/s41598-017-14441-6)
Supplement: Supplementary file 1 — Supplementary information [file 41598_2017_14441_MOESM1_ESM.doc]

# For submission to *Scientific Reports*

# Species composition and seasonal dynamics of aphid parasitoids and hyperparasitoids in wheat fields in northern China

Fan Yang^1^, Lei Xu^1^, Yue-Kun Wu^1^, Qian Wang^1^, Zhi-Wen Yao^1^, Vladimir Žikić^2^, Željko Tomanović^3^, Mar Ferrer-Suay^4^, Jesús Selfa^4^, Juli Pujade-Villar^5^, Michael Traugott^6^, Nicolas Desneux^7^, Yan-Hui Lu^1^, Yu-Yuan Guo^1^

^1^State Key Laboratory for Biology of Plant Diseases and Insect Pests, Institute of Plant Protection, Chinese Academy of Agricultural Sciences, Beijing 100193, China, ^2^Faculty of Sciences and Mathematics, Department of Biology and Ecology, University of Niš, Višegradska 33, 18000 Niš, Serbia, ^3^University of Belgrade, Faculty of Biology, Institute of Zoology, Department of Invertebrate Zoology and Entomology, Belgrade 11000, Serbia, ^4^Universitat de València, Facultat de Ciències Biològiques, Departament de Zoologia, València 46100, Spain, ^5^Universitat de Barcelona, Facultat de Biologia, Departament de Biologia Animal. Avda. Diagonal 645, 08028-Barcelona, Spain. ^6^Mountain Agriculture Research Unit, Institute of Ecology, University of Innsbruck, 6020 Innsbruck, Austria. ^7^INRA (French National Institute for Agricultural Research), Université Côte D’Azur, CNRS, UMR 1355-7254, Institut Sophia Agrobiotech, 400 Route des Chappes, 06903 Sophia Antipolis, France. Correspondence and requests for materials should be addressed to Y.H.L (email: yhlu@ippcaas.cn) and Y.Y.G. (email: yuyuanguo@hotmail.com).

The first two authors contributed equally to this work.

Table S1. The number of parasitoids emerged from the collected mummies in each sampling fields in North China, 2014/2015.

| Year | Location | | No. sampled mummies | No. emerging primary parasitoids | No. emerging hyperparasitoids |
| --- | --- | --- | --- | --- | --- |
|  | Latitude | Longitude |  |  |  |
| 2014 | 39.59902 | 116.53518 | 150 | 2 | 7 |
| 2014 | 39.68511 | 116.48869 | 150 | 0 | 45 |
| 2014 | 39.58176 | 116.4578 | 150 | 6 | 35 |
| 2014 | 39.5003 | 116.40982 | 150 | 0 | 29 |
| 2014 | 39.35098 | 116.21253 | 150 | 39 | 80 |
| 2014 | 39.28614 | 116.26617 | 150 | 37 | 41 |
| 2014 | 39.28157 | 116.19733 | 150 | 50 | 46 |
| 2014 | 39.32435 | 116.1431 | 150 | 66 | 29 |
| 2014 | 39.59406 | 116.78623 | 150 | 5 | 27 |
| 2014 | 39.58709 | 116.84855 | 150 | 21 | 54 |
| 2014 | 39.60612 | 117.10798 | 150 | 13 | 55 |
| 2014 | 39.58749 | 117.06516 | 150 | 5 | 0 |
| 2014 | 39.48722 | 117.07098 | 150 | 47 | 84 |
| 2014 | 39.61212 | 116.9436 | 150 | 61 | 37 |
| 2014 | 39.58455 | 117.06025 | 235 | 54 | 99 |
| 2014 | 39.51129 | 117.09038 | 150 | 6 | 129 |
| 2014 | 39.52858 | 116.93757 | 256 | 39 | 136 |
| 2014 | 39.57221 | 116.92241 | 150 | 9 | 114 |
| 2015 | 39.50198 | 116.455769 | 150 | 31 | 53 |
| 2015 | 39.54358 | 116.48869 | 150 | 39 | 62 |
| 2015 | 39.55701 | 116.347409 | 150 | 34 | 92 |
| 2015 | 39.56649 | 116.276748 | 150 | 31 | 53 |
| 2015 | 39.51204 | 116.264521 | 150 | 19 | 27 |
| 2015 | 39.47506 | 116.151446 | 150 | 44 | 59 |
| 2015 | 39.48011 | 116.082177 | 150 | 54 | 92 |
| 2015 | 39.4429 | 116.080373 | 150 | 38 | 19 |
| 2015 | 39.37667 | 116.044474 | 277 | 100 | 87 |
| 2015 | 39.31819 | 116.04663 | 150 | 70 | 42 |
| 2015 | 39.3757 | 116.158594 | 150 | 62 | 44 |
| 2015 | 39.40945 | 116.128963 | 150 | 31 | 52 |
| 2015 | 39.48722 | 117.07098 | 254 | 62 | 132 |
| 2015 | 39.54566 | 116.499812 | 199 | 68 | 96 |
| 2015 | 39.63084 | 116.481578 | 150 | 13 | 61 |
| 2015 | 39.67074 | 116.642426 | 150 | 9 | 24 |
| 2015 | 39.71574 | 116.724421 | 150 | 32 | 109 |
| 2015 | 39.7298 | 116.788676 | 196 | 86 | 88 |
| 2015 | 39.71929 | 116.859413 | 190 | 57 | 119 |
| 2015 | 39.72805 | 116.988645 | 150 | 50 | 38 |
| 2015 | 39.63263 | 117.025495 | 294 | 119 | 105 |
| 2015 | 39.5185 | 116.850428 | 150 | 28 | 72 |
| 2015 | 39.58137 | 116.977781 | 150 | 35 | 25 |
| 2015 | 39.53957 | 117.019252 | 150 | 32 | 62 |
| 2015 | 39.54978 | 117.090186 | 150 | 21 | 64 |
| 2015 | 39.54736 | 117.147378 | 150 | 27 | 52 |
| 2015 | 39.47726 | 117.06728 | 150 | 10 | 35 |
| 2015 | 39.49931 | 117.015541 | 150 | 21 | 48 |
| 2015 | 39.47163 | 116.920895 | 225 | 20 | 147 |
| 2015 | 39.46717 | 116.857845 | 150 | 33 | 89 |

Table S2. The identification methods of wheat aphid parasitoids collected in North China, 2014/2015.

| Species | Morphological identification | DNA barcoding confirmation |
| --- | --- | --- |
| *Aphidius ervi* Haliday | × | × |
| *Aphidius gifuensis* (Ashmead) | × | × |
| *Aphidius uzbekistanicus* Luzhetski | × | × |
| *Praon volucre* (Haliday) | × | × |
| *Alloxysta fracticornis* (Thomson) | × |  |
| *Alloxysta japonicus* (Ashmead) | × |  |
| *Alloxysta pusilla* (Kieffer) | × |  |
| *Alloxysta victrix* (Westwood) | × |  |
| *Phaenoglyphis villosa* (Hartig) | × | × |
| *Syrphophagus aphidivorus* (Mayr) | × |  |
| *Syrphophagus eliavae* Japoshvili | × |  |
| *Syrphophagus* sp. | × |  |
| *Syrphophagus taeniatus* (Förster) | × |  |
| *Dendrocerus laticeps* (Hedicke) | × | × |
| *Dendrocerus carpenteri* (Curtis) | × | × |
| *Pachyneuron aphidis* (Bouché) | × | × |
| *Asaphes suspensus* (Nees) | × | × |
| *Asaphes vulgaris* Walker | × | × |

Table S3. The amplified sequence number of wheat aphid parasitoids collected in North China, 2014/2015.

| Family | Genus | Species | Level | Target genes | |
| --- | --- | --- | --- | --- | --- |
|  |  |  |  | COI | 16S |
| Braconidae | *Aphidius* | *Aphidius uzbekistanicus* | Primary | 8 | 9 |
| Braconidae | *Aphidius* | *Aphidius ervi* | Primary | 6 | 6 |
| Braconidae | *Aphidius* | *Aphidius gifuensis* | Primary | 7 | 7 |
| Braconidae | *Praon* | *Praon volucre* | Primary | 1 | 1 |
| Figitidae | *Alloxysta* | *Alloxysta consobrina* | Hyper | 0 | 2 |
| Figitidae | *Alloxysta* | *Alloxysta fracticornis* | Hyper | 2 | 1 |
| Figitidae | *Alloxysta* | *Alloxysta japonicus* | Hyper | 3 | 10 |
| Figitidae | *Alloxysta* | *Alloxysta pusilla* | Hyper | 4 | 0 |
| Figitidae | *Alloxysta* | *Alloxysta victrix* | Hyper | 0 | 2 |
| Figitidae | *Phaenoglyphis* | *Phaenoglyphis villosa* | Hyper | 9 | 2 |
| Encyrtidae | *Syrphophagus* | *Syrphophagus aphidivorus* | Hyper | 0 | 0 |
| Encyrtidae | *Syrphophagus* | *Syrphophagus eliavae* | Hyper | 1 | 0 |
| Encyrtidae | *Syrphophagus* | *Syrphophagus* sp. | Hyper | 1 | 0 |
| Encyrtidae | *Syrphophagus* | *Syrphophagus taeniatus* | Hyper | 2 | 0 |
| Megaspilidae | *Dendrocerus* | *Dendrocerus laticeps* | Hyper | 4 | 5 |
| Megaspilidae | *Dendrocerus* | *Dendrocerus carpenteri* | Hyper | 2 | 3 |
| Pteromalidae | *Pachyneuron* | *Pachyneuron aphidis* | Hyper | 10 | 6 |
| Pteromalidae | *Asaphes* | *Asaphes suspensus* | Hyper | 2 | 2 |
| Pteromalidae | *Asaphes* | *Asaphes vulgaris* | Hyper | 8 | 6 |

Table S4. The statistic analysis result of the difference of proportion among different parasitoid taxa that included the two primary parasitoids and various hyperparasitoids on wheat collected from different growing periods in 2015 and 2016.

| **Crop** | **Year** | **Parasitoid groups** | **Sampling Period** | **df** | **Chisq** | **P** |
| --- | --- | --- | --- | --- | --- | --- |
| Wheat | 2015 | Primary vs Hyper | The whole period | 2 | 244.15 | <.0001 |
|  |  |  | Early vs Middle | 1 | 147.47 | <.0001 |
|  |  |  | Early vs Late | 1 | 202.08 | <.0001 |
|  |  |  | Middle vs Late | 1 | 31.17 | <.0001 |
|  |  | Primary | The whole period | 2 | 9.41 | 0.0091 |
|  |  |  | Early vs Middle | 1 | 1.55 | 0.2137 |
|  |  |  | Early vs Late | 1 | 10.21 | 0.0014 |
|  |  |  | Middle vs Late | 1 | 4.42 | 0.0356 |
|  |  | Hyper | The whole period | 26 | 119.72 | <.0001 |
|  |  |  | Early vs Middle | 13 | 78.49 | <.0001 |
|  |  |  | Early vs Late | 12 | 76.39 | <.0001 |
|  |  |  | Middle vs Late | 12 | 22.23 | 0.0350 |
|  | 2016 | Primary vs Hyper | The whole period | 2 | 259.15 | <.0001 |
|  |  |  | Early vs Middle | 1 | 64.88 | <.0001 |
|  |  |  | Early vs Late | 1 | 283.16 | <.0001 |
|  |  |  | Middle vs Late | 1 | 108.78 | <.0001 |
|  |  | Primary | The whole period | 2 | 1.52 | 0.4669 |
|  |  |  | Early vs Middle | 1 | 0.01 | 0.9238 |
|  |  |  | Early vs Late | 1 | 1.30 | 0.2540 |
|  |  |  | Middle vs Late | 1 | 1.40 | 0.2369 |
|  |  | Hyper | The whole period | 22 | 115.78 | <.0001 |
|  |  |  | Early vs Middle | 11 | 45.57 | <.0001 |
|  |  |  | Early vs Late | 11 | 88.58 | <.0001 |
|  |  |  | Middle vs Late | 11 | 39.17 | <.0001 |

Figure S1. The photos of key morphological identification characters of wheat aphid parasitoid species. The photos of *Alloxysta pusilla*, *Pachyneuron aphidis*, *Phaenoglyphis villosa*, *Syrphophagus aphidivorus*, *Syrphophagus eliavae*, *Syrphophagus sp*., *Syrphophagus taeniatus*, *Dendrocerus carpenteri*, *Dendrocerus laticeps*, *Asaphes suspensu*s, *Asaphes vulgaris*, which were also found in cotton fields of northern China, have been showed in our previous article^56^. The parasitoids were morphologically identified using specific identifying characteristics^57-69^.

**(1) *Aphidius uzbekistanicus***


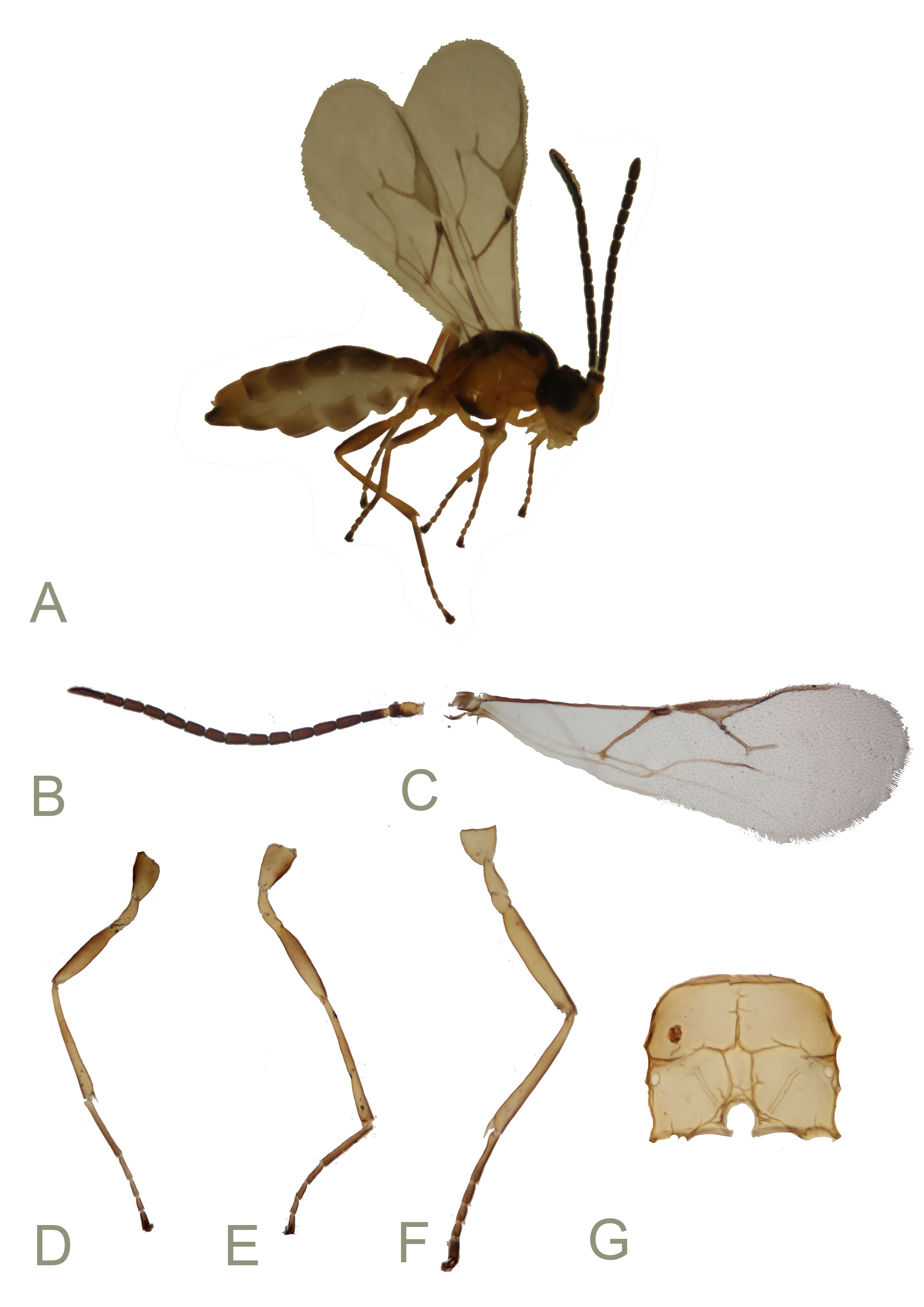


(A) Whole body, (B) antenna, (C) forewing, (D) propodium, (E) mesopodium, (F) metapodium, (G) propodeum

**(2) *Aphidius gifuensis***


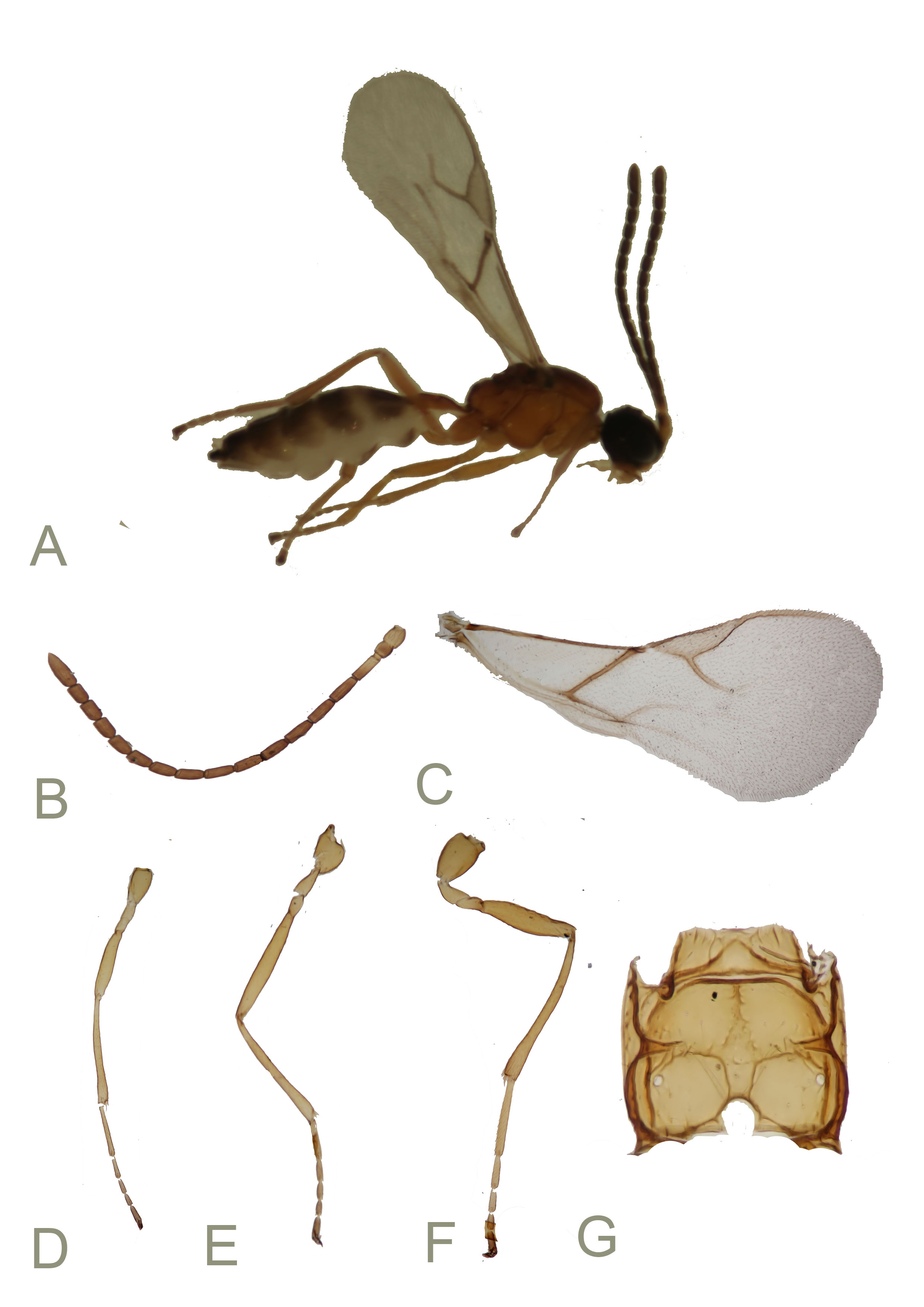


(A) Whole body, (B) antenna, (C) forewing, (D) propodium, (E) mesopodium, (F) metapodium, (G) propodeum.

**(3)** ***Aphidius ervi***


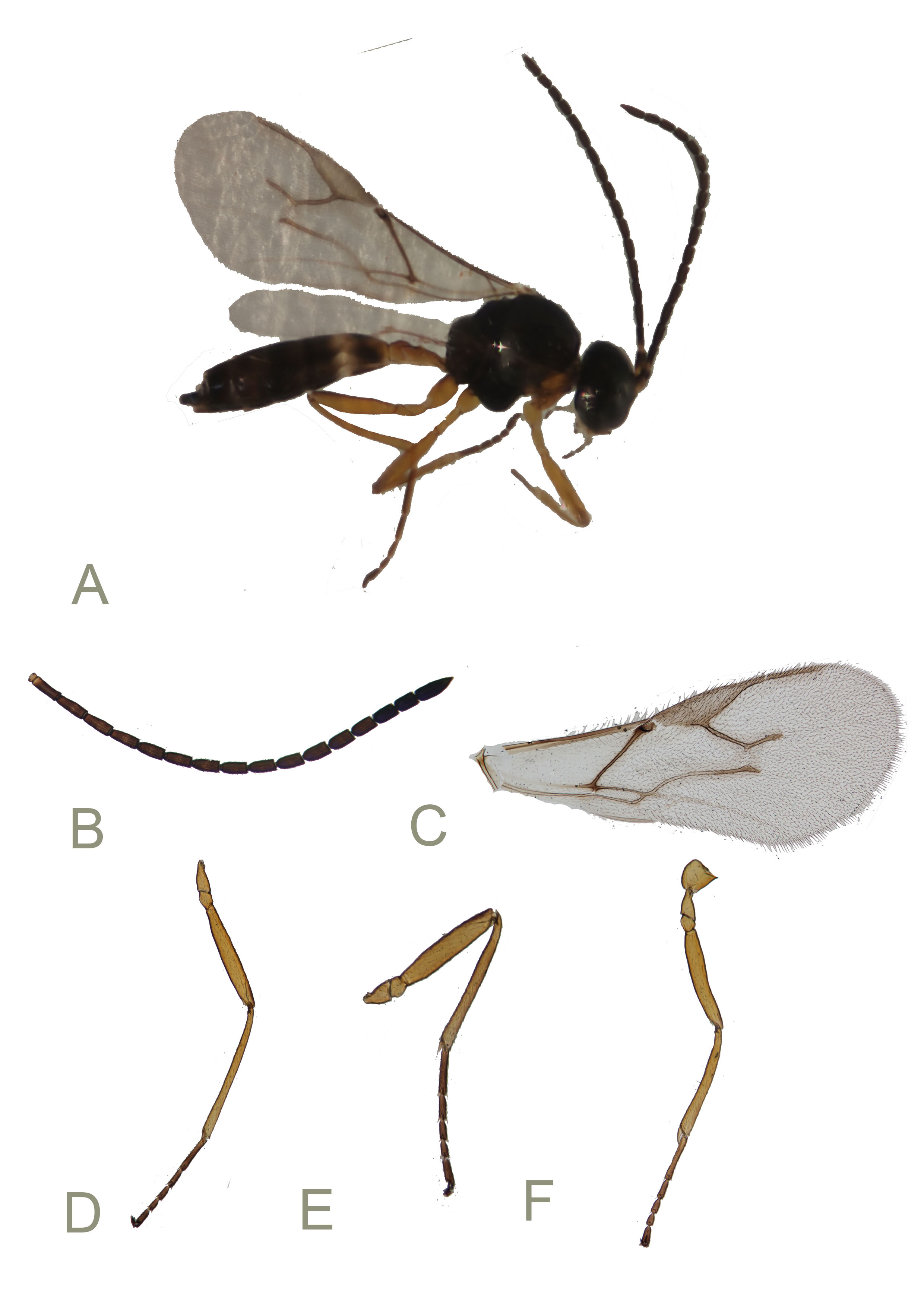


(A) Whole body, (B) antenna, (C) forewing, (D) propodium, (E) mesopodium, (F) metapodium.

**(4) *Alloxysta japonicus***


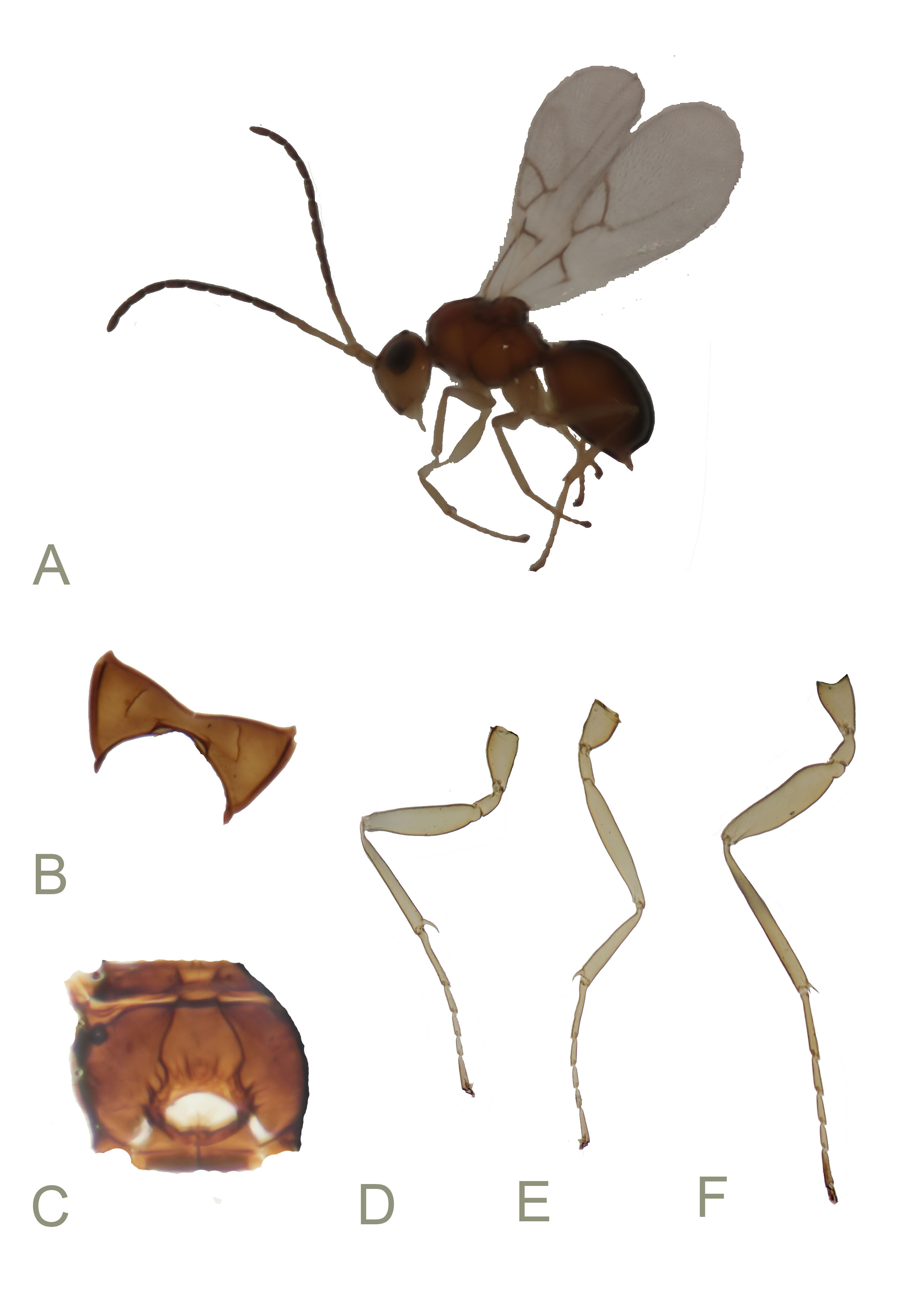


(A) Whole body, (B) pronotum, (C) propodeum, (D) propodium, (E) mesopodium, (F) metapodium.

**References**

56. Yang, F., *et al*. Species composition and richness of aphid parasitoid wasps in cotton fields in northern China. *Sci. Rep.* **7**, 9799 (2017).

57. Doğanlar, M. Morphological studies of the hypopygium and its importance to the taxonomy of the genera *Pachyneuron* and *Euneura* (Hymenoptera: Pteromalidae), with description of a new species of *Pachyneuron* from Turkey. *Fen. Bil. Derg.* **4**, 23-32 (1986).

58. Huang, J. The classification of Aphelinidae (Hymenoptera: Chalcidoidea). Chongqing Publishing Group (In Chinese) (1994).

59. Shi, Z. Y. & Shen, X. C. Parasitoid identification. Chinese Agricultural Science and Technology Press (In Chinese) (1995).

60. Gibson, G. A. P. & Vikberg, V. The species of Asaphes Walker from America north of Mexico, with remarks on extralimital distributions of taxa (Hymenoptera: Chalcidoidea, Pteromalidae). *J. Hymenopt. Res.* **7**, 209-256 (1998).

61. Xiao, H. & Huang, D. W. A taxonomic study on Asaphes (Hymenoptera: Pteromalidae) from China, with descriptions of four new species. *Entomologia Sinica* **7**, 193-202 (2000).

62. Alekseev, V. N. & Radchenko, T. D. Ceraphronoid wasps (Hymenoptera, Ceraphronoidea) of the fauna of the Ukraine-Communication 1[J]. *Vestnik zoologii* **35**, 3-16 (2001).

63. Chen, J. H. & Shi, Q. X. Systematic studies on aphidiidae of China (Hymenoptera: Aphidiidae). Fujian Science & Technology Publishing House (In Chinese) (2001).

64. Gibson, G. A. P. The Australian species of Pachyneuron Walker (Hymenoptera: Chalcidoidea: Pteromalidae). *J. Hymenopt. Res.* **10**, 29-54 (2001).

65. Japoshvili, G. New data on species of Syrphophagus (Hymenoptera: Encyrtidae) from Transcaucasia and Turkey. *Ann. Entomol. Soc. Am.* **100**, 683-687 (2007).

66. Xiao, H., Jiao, T. Y. & Huang, D. W. Pachyneuron (Hymenoptera: Pteromalidae) from China. *Orient. Insects* **43**, 341-359 (2009).

67.Rakhshani, E., *et al*. Parasitoids (Hymenoptera: Braconidae: Aphidiinae) of northeastern Iran: Aphidiine-aphid-plant associations, key and description of a new species. *J. Insect Sci.* **12**, 43 (2012).

68. Ferrer-Suay, M., Selfa, J. & Pujade-Villar, J., Charipinae fauna (Hymenoptera: Figitidae) from Asia with a description of 11 new species. *Zool. Stu.* **52**, 41 (2013a).

69. Ferrer-Suay M, *et al*. A contribution to the knowledge of Charipinae (Hymenoptera: Cynipoidea: Figitidae) associated with aphids (Hemiptera: Aphididae) from Iran, including new records. *North-West J. Zool.* **9**, 30-44 (2013b).
